# Longitudinal Study of the Impact of Community Belonging on Memory in Older Adults Residing in Life Plan Communities

**DOI:** 10.1093/geroni/igaf122.504

**Published:** 2025-12-31

**Authors:** Renae Smith-Ray, Jennifer Smith, Laurel Mertz

**Affiliations:** Mather Institute, Evanston, Illinois, United States; Mather Institute, Evanston, Illinois, United States; Mather Institute, Evanston, Illinois, United States

## Abstract

Evidence suggests that social well-being, including community belonging, is an important predictor of health. Older adults with strong community belonging (i.e., feeling of connection to one’s proximal places, groups, and individuals) consistently demonstrate better health outcomes compared to those with lower belonging. There is less known about the impact of belonging on older adults who reside in Life Plan Communities (LPCs). The purpose of this study was to examine the impact of belonging on memory over three years (2018-2020) among a cohort of older adults residing in LPCs. The longitudinal cohort design entailed surveying participants annually, including measures of community belonging and self-reported memory. Participants (N = 8,070) had a mean age of 83.9 years, were 67.2% female, and an average length of LPC residence of 6 years. A linear mixed model with repeated measures was used to examine the impact of belonging on memory over time. The analysis revealed significant main effects of belonging, F(1,14446.8) = 193.4, p < 0.001, and year, F(2, 5720.6)=3.43, p = 0.032, indicating that higher belonging was associated with better memory and that memory varied across years. There was also a significant interaction between year and belonging, F(2,5681.8) = 4.7, p = 0.010, indicating that the change in memory scores over time differed by participants’ belonging score. Post-hoc tests revealed that memory declined over time, but the decline was slightly moderated by belonging. These results suggest that, for older adults residing in LPCs, greater community belonging is associated with better memory over time, underscoring the importance of belonging on cognitive health.

